# In vivo biodistribution and pharmacokinetics of sotrovimab, a SARS-CoV-2 monoclonal antibody, in healthy cynomolgus monkeys

**DOI:** 10.1007/s00259-022-06012-3

**Published:** 2022-10-28

**Authors:** Tolulope A. Aweda, Shih-Hsun Cheng, Stephen C. Lenhard, Armin Sepp, Tinamarie Skedzielewski, Chih-Yang Hsu, Shelly Marshall, Heather Haag, Jonathan Kehler, Prabhas Jagdale, Alessia Peter, Michael A. Schmid, Andrew Gehman, Minh Doan, Andrew P. Mayer, Peter Gorycki, Marie Fanget, Christophe Colas, Brenda Smith, Curtis C. Maier, Hasan Alsaid

**Affiliations:** 1 Bioimaging, GSK, 1250 S. Collegeville Rd, Collegeville, PA 19426 USA; 2 Certara UK Ltd, Sheffield, UK; 3 Integrated Biological Platform Sciences, GSK, Collegeville, PA USA; 4 Bioanalysis, Immunogenicity & Biomarkers, GSK, Collegeville, PA USA; 5 DMPK, GSK, Collegeville, PA USA; 6 mAb Engineering & Bioanalytics, Humabs BioMed SA, Vir Biotechnology, Inc, Bellinzona, Switzerland; 7 Non-Clinical and Translational Statistics, GSK, Collegeville, PA USA; 8 Bioanalytical Department, Vir Biotechnology, Inc, San Francisco, CA USA; 9 DMPK, Vir Biotechnology, Inc, San Francisco, CA USA; 10 Toxicology, Vir Biotechnology, Inc, San Francisco, CA USA; 11 Non-Clinical Safety, GSK, Collegeville, PA USA

**Keywords:** SARS-CoV-2, Sotrovimab, Monoclonal antibody, Respiratory tract, Biodistribution, ^89^Zr-PET, Physiologically based pharmacokinetics (PBPK), Radiomics

## Abstract

**Purpose:**

Sotrovimab (VIR-7831), a human IgG1κ monoclonal antibody (mAb), binds to a conserved epitope on the SARS-CoV-2 spike protein receptor binding domain (RBD). The Fc region of VIR-7831 contains an LS modification to promote neonatal Fc receptor (FcRn)–mediated recycling and extend its serum half-life. Here, we aimed to evaluate the impact of the LS modification on tissue biodistribution, by comparing VIR-7831 to its non-LS-modified equivalent, VIR-7831-WT, in cynomolgus monkeys.

**Methods:**

^89^Zr-based PET/CT imaging of VIR-7831 and VIR-7831-WT was performed up to 14 days post injection. All major organs were analyzed for absolute concentration as well as tissue:blood ratios, with the focus on the respiratory tract, and a physiologically based pharmacokinetics (PBPK) model was used to evaluate the tissue biodistribution kinetics. Radiomics features were also extracted from the PET images and SUV values.

**Results:**

SUV_mean_ uptake in the pulmonary bronchi for ^89^Zr-VIR-7831 was statistically higher than for ^89^Zr-VIR-7831-WT at days 6 (3.43 ± 0.55 and 2.59 ± 0.38, respectively) and 10 (2.66 ± 0.32 and 2.15 ± 0.18, respectively), while the reverse was observed in the liver at days 6 (5.14 ± 0.80 and 8.63 ± 0.89, respectively), 10 (4.52 ± 0.59 and 7.73 ± 0.66, respectively), and 14 (4.95 ± 0.65 and 7.94 ± 0.54, respectively). Though the calculated terminal half-life was 21.3 ± 3.0 days for VIR-7831 and 16.5 ± 1.1 days for VIR-7831-WT, no consistent differences were observed in the tissue:blood ratios between the antibodies except in the liver. While the lung:blood SUV_mean_ uptake ratio for both mAbs was 0.25 on day 3, the PBPK model predicted the total lung tissue and the interstitial space to serum ratio to be 0.31 and 0.55, respectively. Radiomics analysis showed VIR-7831 had mean-centralized PET SUV distribution in the lung and liver, indicating more uniform uptake than VIR-7831-WT.

**Conclusion:**

The half-life extended VIR-7831 remained in circulation longer than VIR-7831-WT, consistent with enhanced FcRn binding, while the tissue:blood concentration ratios in most tissues for both drugs remained statistically indistinguishable throughout the course of the experiment. In the bronchiolar region, a higher concentration of ^89^Zr-VIR-7831 was detected. The data also allow unparalleled insight into tissue distribution and elimination kinetics of mAbs that can guide future biologic drug discovery efforts, while the residualizing nature of the ^89^Zr label sheds light on the sites of antibody catabolism.

**Supplementary Information:**

The online version contains supplementary material available at 10.1007/s00259-022-06012-3.

## Introduction

Monoclonal antibody (mAb) serum half-life extension is a strategy that is increasingly employed in next-generation mAb biologics to allow for lower doses or reduced dosing frequency [1]. While the impact of Fc region engineering on plasma clearance is well documented across species, the consequences for tissue distribution and its time course are largely unknown despite its significance to the tissue-embedded target engagement. It has been shown in several studies that enhanced association of the IgG Fc region to the neonatal Fc receptor (FcRn) can prolong the half-life of the antibody [1–3]. The interaction with FcRn leads to partial recycling of endocytosed IgG by rerouting the antibodies back to the cell surface rather than toward lysosomal degradation [4]. The interaction with FcRn is pH-dependent: endocytosed IgGs bind to FcRn in the slightly acidic (pH < 6.5) environment of late endosomes but not at physiological pH = 7.4, resulting in bound IgG trafficking to the cell surface and extracellular release [5]. A number of modifications on the Fc region have been demonstrated to increase the affinity to FcRn at low pH, thereby modulating the pharmacokinetics of the antibody, increasing serum availability, and prolonging therapeutic effect [1, 6, 7]. One of these Fc variants is the LS modification which comprises of amino acid substitutions (Met428Leu + Asn434Ser), and it was shown to prolong the serum half-lives of antibodies up to 3 – 5-fold [8].

Sotrovimab (VIR-7831) is an LS-modified human IgG1κ mAb that binds to a highly conserved epitope on the severe acute respiratory syndrome coronavirus 2 (SARS-CoV-2) spike protein, neutralizing the virus [9]. VIR-7831 is effective for the treatment of mild-to-moderate coronavirus disease 2019 (COVID-19), the disease caused by SARS-CoV-2 [10]. Previous studies in monkeys with an anti-HIV mAb, VRC01, demonstrated that the LS modification increased half-life and enhanced rectal mucosal tissue localization compared to wild-type VRC01. It also afforded superior protection from rectal simian-HIV infection [11]. Since SARS-CoV-2 is primarily a respiratory virus, it was desirable to understand if the LS modification in VIR-7831 afforded similar advantages for lung biodistribution.

Positron emission tomography and computed tomography (PET/CT) has been extensively used to study the tissue distribution of monoclonal antibodies labeled with zirconium-89 (^89^Zr) which allows for the full body monitoring of drug distribution in live animals for a long duration post-injection as a result of its 78.42-h half-life [12, 13]. In this study, PET/CT imaging was conducted in cynomolgus monkeys to determine the potential effects of the LS modification on biodistribution of VIR-7831 in all major organs, with special focus on the upper and lower respiratory tract. Drug concentrations in blood, tissues, and PET data were modeled by physiologically based pharmacokinetics (PBPK) to evaluate the tissue distribution kinetics of VIR-7831 and the non-LS-modified wild-type VIR-7831-WT antibodies.

To further investigate differences between the groups, radiomics analysis was conducted [14], involving the extraction and selection of high-dimensional quantitative image features such as intensity profiles, shapes, and textures. Additionally, to analyze the spatial distribution of ^89^Zr-labeled mAb with proximity to blood supply, the lung was further separated into three regions using levelset analysis based on the distance to blood supply.

## Materials and methods

### Radiolabeling and binding activity of ^89^Zr-labeled mAbs

VIR-7831 and VIR-7831-WT (GSK) were modified with p-SCN-Bn-deferoxamine (DFO) (Macrocyclics) at an input antibody to chelate molar ratio of 1:1; then, ^89^Zr was complexed with the obtained DFO-labeled mAb at a ratio of 111 MBq/mg of mAb. The stability of ^89^Zr-mAbs was tested by incubation in 10% monkey serum and in PBS at 37 °C for up to 7 days.

The binding affinities of VIR-7831 and VIR-7831-WT for the human FcRn and the SARS-CoV-2 spike protein receptor binding domain (RBD) were determined by ELISA (enzyme-linked immunosorbent assay) before and after radiolabeling to confirm that the radiolabeling procedure with ^89^Zr did not affect the functionality of these antibodies (see the Supplemental Materials).

### Experimental design and PET/CT imaging

Minimally sufficient group sizes of *n* = 3 naïve female cynomolgus monkeys (3.5–5.5 years old) from Envigo, USA, were used in this study. Paired animals were assigned to the different groups based on body weight. The study was conducted in accordance with the GSK Policy on the Care, Welfare, and Treatment of Laboratory Animals and was reviewed by the Institutional Animal Care and Use Committee at GSK. Each monkey had an intravenous catheter placed in either the tail vein or saphenous vein for injection of 1 mg (~ 92.5 MBq, 2.5 mCi) radiolabeled mAb mixed with unlabeled mAb for a total dose of 5 mg/kg in approximately 10 mL total volume (Supplemental Table S1). In this study, the 5 mg/kg dose replicates what was used in previous pharmacokinetic studies in this species and represents a lower exposure than observed clinically at 500 mg, a dose that was demonstrated to provide protective exposures against susceptible variants in the lung for up to 28 days [10]. The dose was administered by IV slow bolus injection over 5 min on day 0. PET/CT acquisition was performed on days 1, 3, 6, 10, and 14 using a Mediso MultiScan LFER150 system. Details are in the Supplemental Material.

Quantitative standardized uptake value (SUV_mean_) of ^89^Zr-mAb was measured using VivoQuant software v.3.5 (InviCRO) in the organs and tissues of interest (brain, nasal cavity, pharynx, larynx, trachea, pulmonary bronchi, total lungs including “air space,” liver, myocardium, “left ventricular, LV,” blood in left ventricular, spleen, renal cortex, renal medulla, small intestine, large intestine, bladder, axillary lymph node, and muscle). ^89^Zr-mAb uptake in the lung tissue (excluding air space) was calculated using a correction factor of 2.81 [15]. Data are reported as group mean ± SEM (standard error of mean).

### Drug concentration in tissues, blood, and serum from imaging

Absolute molar concentrations of ^89^Zr-labeled VIR-7831 and VIR-7831-WT in organs of interest were calculated using the specific activity *a*(Bq/mole) of the dose as given to each animal (Supplemental Material) [16].

Ex vivo blood radioactivity was measured on samples collected before each imaging session by gamma counting using a Wizard 2480 Gamma Counter (PerkinElmer) and corrected for ^89^Zr decay. The serum concentration was calculated from the blood concentration using a monkey hematocrit correction factor of 0.38 [16]. Total serum concentrations (both labeled and unlabeled antibody) were measured by Gyrolab immunoassay for all animals up to day 14, which was extended for animals # 3–6 up to week 8.

### Statistical analysis

The statistical analysis software SAS (version 9.4 TS Level 1 M) was used to model the PET SUV_mean_ and PET tissue:blood ratio data values and to perform statistical tests of mAb group differences at a 0.05 level of significance.

Terminal half-lives for VIR-7831-WT and VIR-7831 were calculated from the day 6 to week 8 serum immunoassay data with a single-exponential decay model by non-linear regression in Matlab 2019a/SimBiology v5.8.4 (MathWorks).

Quantitative tissue distribution of VIR-7831 and VIR-7831-WT was analyzed in terms of absolute concentrations within the framework of cross-species/cross-modality PBPK as described previously [13, 17]. Details are in the Supplemental Material.

### Advanced image analysis

Radiomics feature analysis was used to distinguish image patterns between the different groups to derive more insights into the PET distribution [14, 18]. Levelset analysis of the lung using scikit-fmm [19] was performed to investigate SUV distribution with proximity to major blood supply. Details are in the Supplemental Material.

## Results

### Quality and functionality of ^89^Zr mAbs

DFO-VIR-7831 and DFO-VIR-7831-WT antibody conjugates were successfully prepared and purity was confirmed by SEC HPLC. Radiolabeling yield for the ^89^Zr-DFO antibodies before purification through a size exclusion cartridge was 84 ± 3%, while ≥ 95% radiochemical purity was achieved after SEC purification as confirmed by analytical HPLC. Molar activities (specific activity) of 15.9 ± 1.2 MBq/nmol (2.87 ± 0.2 mCi/mg) and 13.1 ± 1.1 MBq/nmol (2.36 ± 0.2 mCi/mg) were achieved for ^89^Zr-VIR-7831 and ^89^Zr-VIR-7831-WT, respectively. Both ^89^Zr-labeled antibody conjugates showed good stability in both buffer and 10% monkey serum with less than 15% loss of ^89^Zr after 7 days (Supplemental Fig. S1).

**Fig. 1:**
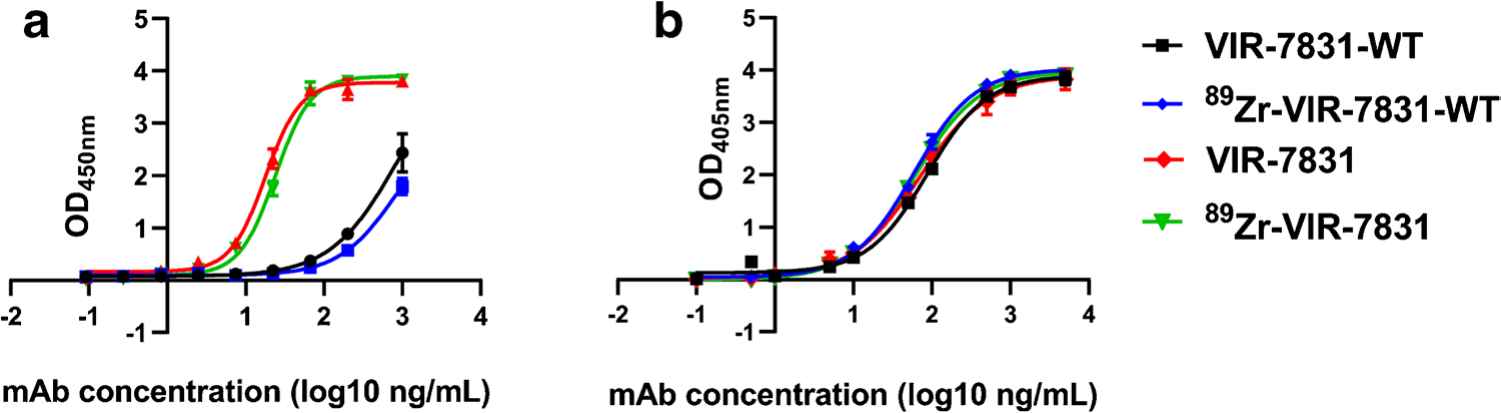
FcRn (**a**) and RBD (**b**) ELISA binding curves of VIR-7831-WT, 89Zr-VIR-7831-WT, VIR-7831, and 89Zr-VIR-7831 obtained at room temperature and at pH = 6 with freshly labeled antibodies

The FcRn binding curve (Fig. 1a) showed stronger binding of FcRn with VIR-7831 than with VIR-7831-WT at pH = 6. Furthermore, the FcRn binding curves obtained with the radiolabeled antibodies (^89^Zr-VIR-7831 and ^89^Zr-VIR-7831-WT) overlapped with the ones for the non-radiolabeled VIR-7831 and VIR-7831-WT, respectively. This data shows no changes in FcRn binding after radiolabeling with ^89^Zr. The binding curve of the antibodies to the RBD (Fig. 1b) showed excellent overlap between VIR-7831 and VIR-7831-WT antibodies. Furthermore, the curves obtained with the radiolabeled antibodies (^89^Zr-VIR-7831 and ^89^Zr-VIR-7831-WT) overlapped with the ones for the non-radiolabeled VIR-7831 and VIR-7831-WT, respectively. This shows that the radiolabeling had no effect on the RBD binding of both antibodies.

### In vivo PET biodistribution and quantification of ^89^Zr-mAbs

**Fig. 2:**
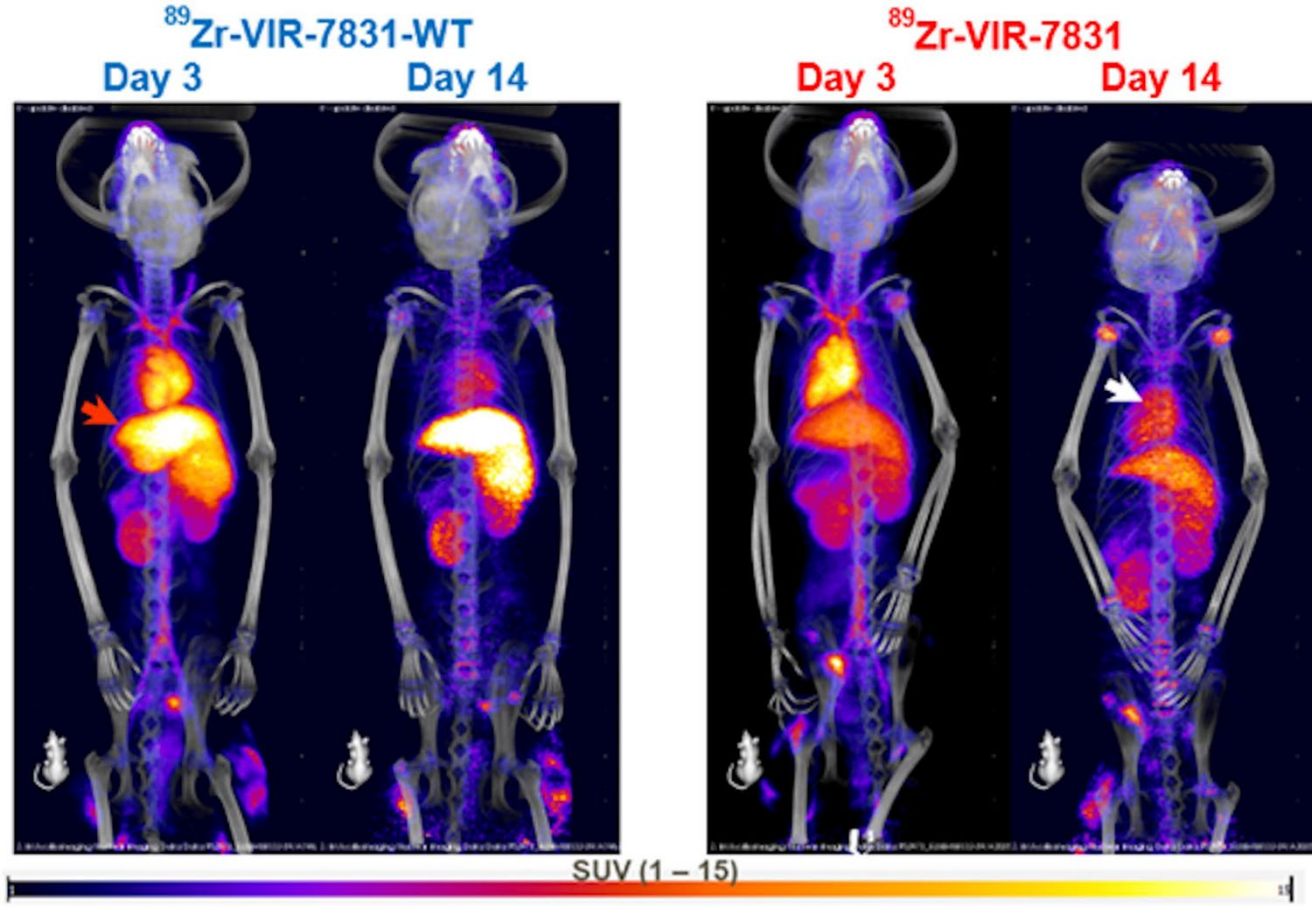
Representative 3D co-registered PET/CT (maximum intensity projection) images of cynomolgus monkeys on days 3 and 14 post-injection with either ^89^Zr-VIR-7831 or ^89^Zr-VIR-7831-WT. Red and white arrows indicate the liver and heart, respectively

**Fig. 3:**
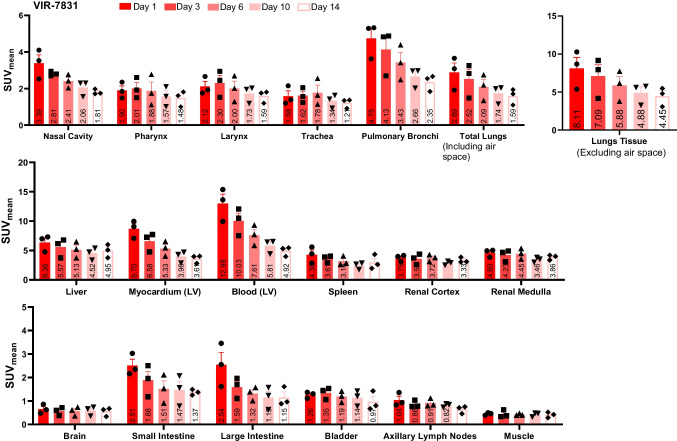
Group mean from PET signal showing ^89^Zr-VIR-7831 uptake (SUV_mean_) measured on days 1, 3, 6, 10, and 14 following a single 5 mg/kg dose of VIR-7831

**Fig. 4:**
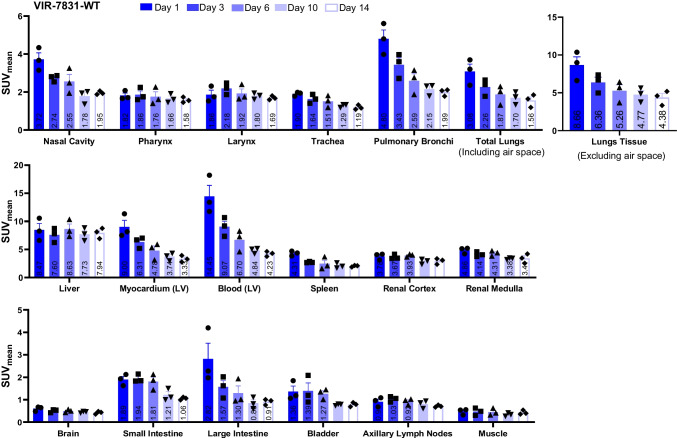
Group mean from PET signal showing ^89^Zr-VIR-7831-WT uptake (SUV_mean_) measured on days 1, 3, 6, 10, and 14 following a single 5 mg/kg dose of VIR-7831-WT

Figure 2 shows representative PET/CT images of cynomolgus monkeys on days 3 and 14 post-injection with either VIR-7831 or VIR-7831-WT. The images show higher uptake of ^89^Zr-VIR-7831-WT in the liver compared to ^89^Zr-VIR-7831 on day 3 and day 14, and a higher blood uptake in the heart for ^89^Zr-VIR-7831 compared to ^89^Zr-VIR-7831-WT on day 14. The quantitative group mean uptake (SUV_mean_) data of ^89^Zr-VIR-7831 and ^89^Zr-VIR-7831-WT for all tissues and at all time points are shown in Fig. 3 and Fig. 4, respectively. A summary of the mean uptake (SUV_mean_) for tissues showing statistical differences between antibodies is shown in Table 1. The SUV_mean_ after day 1 was trending higher in the blood (left ventricular) in the VIR-7831-treated group compared to the VIR-7831-WT with statistical significance achieved on day 10 (5.81 ± 0.69 and 4.84 ± 0.29, respectively; *p* = 0.0375). There was a trend of higher group mean ^89^Zr-VIR-7831 uptake in the pulmonary bronchi compared to ^89^Zr-VIR-7831-WT uptake beginning on day 3 that achieved statistical significance on day 6 (3.43 ± 0.55 and 2.59 ± 0.38, respectively) and on day 10 (2.66 ± 0.32 and 2.15 ± 0.18, respectively). Also, ^89^Zr-VIR-7831 uptake in the liver compared to ^89^Zr-VIR-7831-WT uptake was statistically lower on day 6 (5.14 ± 0.80 and 8.63 ± 0.89, respectively), on day 10 (4.52 ± 0.59 and 7.73 ± 0.66, respectively), and on day 14 (4.95 ± 0.65 and 7.94 ± 0.54, respectively). No other noteworthy differences between ^89^Zr-VIR-7831 uptake and ^89^Zr-VIR-7831-WT uptake were detected in the examined tissues.

**Table 1:** Summary table for average of SUV_mean_ in tissues with *p* values between VIR-7831 and VIR-7831-WT groups. Significant statistical differences at *p* < 0.05

Organ	Day post-injection	VIR-7831 SUV_mean_	VIR-7831-WT SUV_mean_	*p* value
Blood (LV)	1	12.98	14.45	0.3674
	3	10.03	9.07	0.1817
	6	7.61	6.70	0.0720
	10	5.81	4.84	0.0375
	14	4.92	4.23	0.0668
Pulmonary bronchi	1	4.75	4.80	0.8917
	3	4.13	3.43	0.0523
	6	3.43	2.59	0.0080
	10	2.66	2.15	0.0262
	14	2.35	2.07	0.0609
Liver	1	6.36	8.47	0.1001
	3	5.57	7.60	0.0715
	6	5.13	8.63	0.0233
	10	4.52	7.73	0.0218
	14	4.95	7.94	0.0293

The tissue:blood SUV_mean_ ratios calculated for ^89^Zr-VIR-7831 and ^89^Zr-VIR-7831-WT increased with time during the 2-week imaging study, as shown in Supplemental Fig. S2 and Fig. S3. Tissue:blood ratios in the liver were significantly lower for ^89^Zr-VIR-7831 than for ^89^Zr-VIR-7831-WT at all time points. While there were occasional statistically significant differences in tissue:blood ratios observed in other tissues, these findings were considered sporadic because they were not consistent over time or in directionality.

On day 3, which is expected to roughly correspond to the end of the antibody distribution phase [20], the ^89^Zr-VIR-7831 group mean tissue:blood ratio in the pulmonary bronchi (0.41) was higher than in other respiratory tract tissues, including the nasal cavity (0.29), pharynx (0.20), larynx (0.23), trachea (0.16), and total lung (0.25, including air space). ^89^Zr-VIR-7831 tissue:blood ratio in the lung tissue excluding air space was 0.70.

### Serum drug concentration

**Fig. 5:**
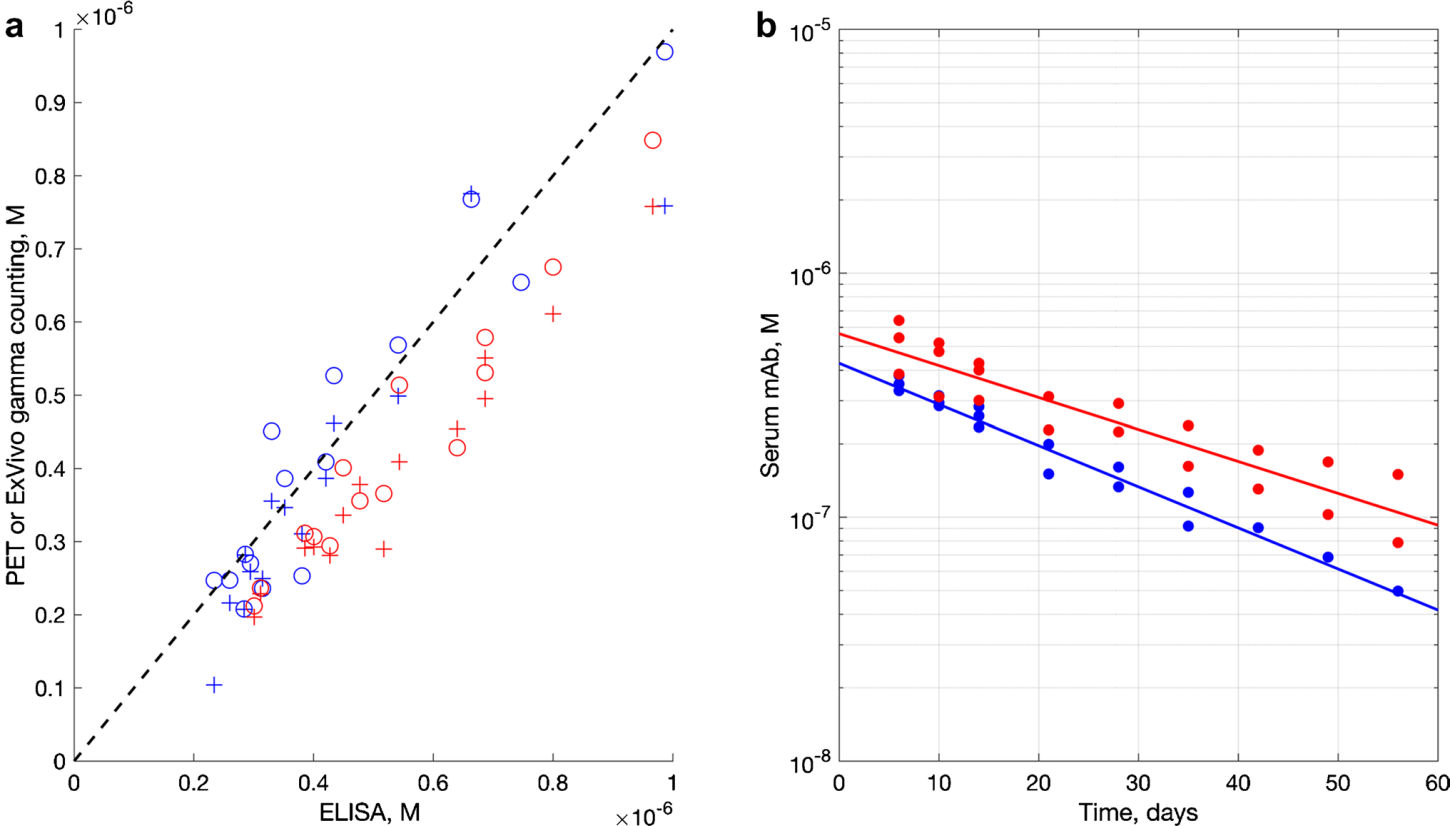
Monoclonal antibody, mAb concentration (**a**) as measured by ex vivo gamma counting (circles) and PET (crosses) vs ELISA for VIR-7831-WT (blue) and VIR-7831 (red). Dotted line: linear proportional prediction. Serum half-life (**b**) of VIR-7831 (red) and VIR-7831-WT (blue) as measured by Gyrolab immunoassay. Terminal half-life was determined from days 6 to 56 data

Drug concentration profiles derived from the analysis of the labeled fraction of the dosed mAb from the left ventricular PET and the ex vivo gamma counting showed very good agreement with the data from the Gyrolab immunoassay of VIR-7831 and VIR-7831-WT measuring total antibody concentrations as shown on Fig. 5a. The calculated terminal half-life is 21.3 ± 3.0 days for VIR-7831 and 16.5 ± 1.1 days for VIR-7831-WT. The fitted data are shown on Fig. 5b. Serum concentration data from animal #4 receiving VIR-7831-WT demonstrated increasing deviation from linearity over the last three data points (from 42 days onward). Therefore, full time course immunoassay data was available only for one VIR-7831-WT-treated animal (#6) and two VIR-7831-treated animals (#3 and #5) after 42 days.

### Tissue concentrations based on PBPK modeling

**Fig. 6:**
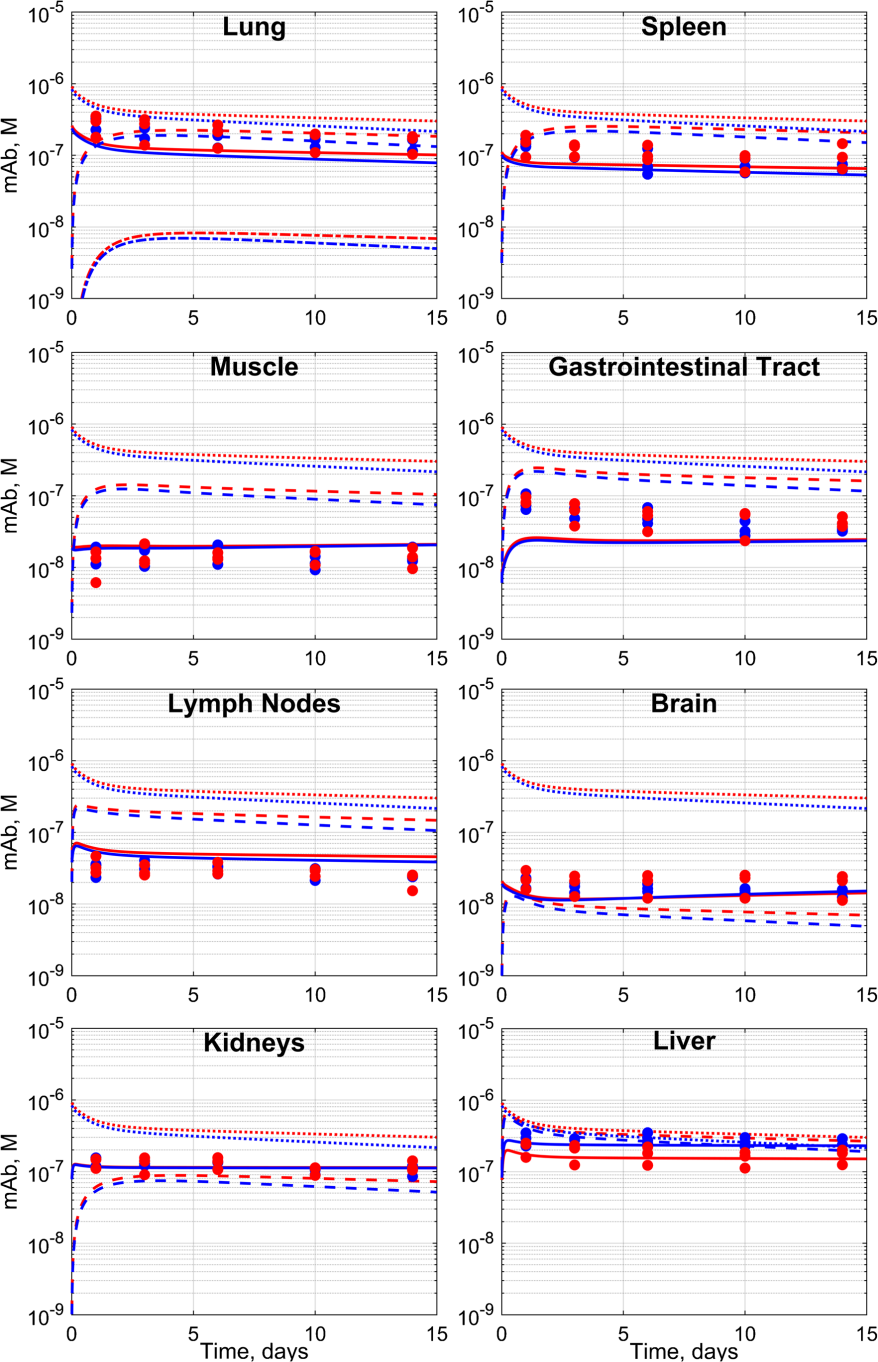
The observed and physiologically based pharmacokinetics (PBPK)–predicted total tissue concentrations with no model adjustment for the lungs, spleen, skeletal muscle, gastrointestinal tract, lymph nodes, brain, kidney, and liver. Red and blue fits represent VIR-7831 and VIR-7831-WT mAb respectively. The solid line (–) fits total mAb in the tissue, the dashed line (----) fits mAb in the interstitial space, and the dotted line (….) fits mAb in serum while solid dots are the experimental values for ^89^Zr antibodies in organs. In the lungs, the dash-dotted line denotes the predicted alveolar epithelial lining fluid concentration

Further quantitative mechanistic insight into VIR-7831-WT and VIR-7831 tissue distribution was sought by PBPK to model the tissue penetration kinetics of VIR-7831-WT and VIR-7831 mAbs and the contribution of ^89^Zr residualization from catabolized mAbs to the overall detected signal. The default physiological system parameters [13] provided satisfactory fitting to the SUV measurements for the lungs, skeletal muscle, spleen, brain, lymph nodes, and gastrointestinal tract with no parameter adjustment, as shown on Fig. 6.

The PBPK model predicted higher concentrations of VIR-7831 vs VIR-7831-WT in the total lung tissue, the interstitial space, and the alveolar epithelial lining fluid (125 nM vs 109 nM, 219 nM vs 188 nM, 8 nM vs 7 nM on day 3, respectively) (Supplemental Table S2). However, tissue:serum ratios were similar up to day 14 (0.31, 0.55, and 0.02 on day 3, respectively) (Supplemental Table S3). In the case of the kidneys and liver, the observed signal was stable in time and substantially exceeded the default PBPK model prediction and therefore, additional modeling was performed. The kidney signal matched the model if it was assumed that 1.1% of the injected ^89^Zr was not bound to the mAb. For the liver, satisfactory agreement between the data and the model was achieved by non-linear least squares optimization of the amount of the dose that was irreversibly captured in the organ during the first 24 h post-dosing.

### Predictive radiomics features and levelset analysis

**Fig. 7:**
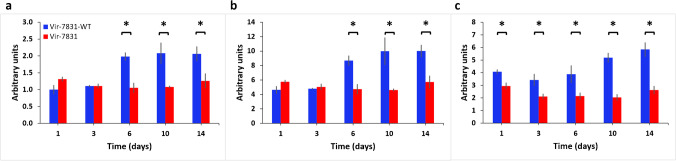
Predictive PET intensity features in the lungs and liver that distinguishes the two groups. (**a**) PET skewness at days 6, 10, and 14 shows significant difference between the two groups VIR-7831 and VIR-7831-WT. (**b**) PET kurtosis at days 6, 10, and 14 shows significant difference between the two groups. (**c**) Liver PET intensity interquartile range at all time points significantly distinguishes the two groups. *p < 0.05

Radiomics analysis detected differences only in the lung and liver and no other ROI. Skewness of the PET intensity histogram inside the lungs was significantly different between antibodies using the linear mixed effect model (*p* < 0.05) between the two groups at days 6, 10, and 14. The VIR-7831-WT group had a longer right tail compared to the VIR-7831 group as shown in Fig. 7a. Skewness measures tail length of the intensity histogram with positive values indicating right tail and negative values indicating left tail. Also, kurtosis of the PET intensity histogram inside the lungs was significantly different (*p* < 0.05) between the two groups at days 6, 10, and 14. Kurtosis measures the spread of the intensity histogram, with high values indicating toward the tails and low values indicating toward the mean. The VIR-7831-WT group had higher kurtosis with distribution more toward the tails while the VIR-7831 group was more centralized toward the mean as shown in Fig. 7b.

Combining both features, VIR-7831 had more voxels with PET intensity similar to the mean indicating more uniform uptake. Similar observation can be seen in the liver with interquartile range; VIR-7831 consistently had lower interquartile range with significant difference at all time points implying more uniform uptake as shown in Fig. 7c. Interquartile range measures the difference between the 75 and 25 percentiles of the intensity histogram. Larger numbers indicate wider spread while smaller numbers indicate more centralized distribution.

**Fig. 8:**
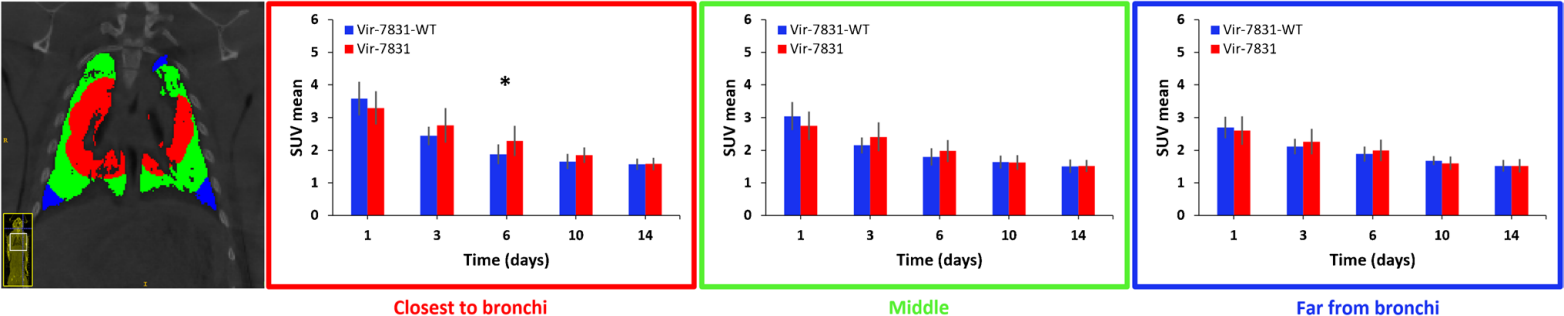
3D levelset analysis with manually chosen distances classified the lung into three groups based on their distance to the bronchi (near, intermediate, far). Average SUV_mean_ within each group were compared over the five time points. Statistical significance between VIR-7831 and VIR-7831-WT was observed at day 6 for the region closest to the bronchi. *p < 0.05

The 3D levelset analysis of the lung shown in Fig. 8 displays the average of SUV_mean_ in the three distance groups across all time points. The VIR-7831 group had statistically higher uptake in the region closest to the bronchi on day 6 (*p* < 0.05) and reduced to similar levels as VIR-7831-WT on days 10 and 14. The VIR-7831 group tended to decay slower compared to the VIR-7831-WT group.

## Discussion

This study utilized PET imaging to evaluate tissue distribution of a radiolabeled LS-modified anti-SARS-CoV-2 IgG1 mAb compared to that of its wild-type counterpart. This publication presents the quantitative and mechanistic insights into tissue distribution and catabolism of biologics that can be obtained from PET/CT imaging. The LS modification comprises amino acid substitutions known to enhance affinity of the antibody for the human FcRn [8]. We verified that the stronger binding of VIR-7831 for the human FcRn at acidic pH was not affected by the ^89^Zr labeling procedure. This potentially translated to longer half-life exhibited by VIR-7831 as compared to VIR-7831-WT (21.3 ± 3.0 days vs 16.5 ± 1.1 days, respectively). However, FcRn-binding affinity is not the sole factor that determines the half-life of antibodies even in the absence of target-mediated clearance since the impact from the Fab regions can also have an effect though previously insufficiently understood [21]. The terminal 16.5-day half-life of VIR-7831-WT is more than double of that of typical human mAbs in monkeys (≈7.5 days) [20], while the 21.3-day half-life of VIR-7831 is similar to that of an half-life extended anti-VEGF mAb (albeit one carrying different set of Fc mutations) [22]. Overall, antibody half-life correlation from FcRn affinity is moderate, implicating dependence on other factors [23]. Therefore, the modest half-life extension of VIR-7831 in monkeys primarily reflects atypically and unexpectedly long half-life of the parent wild-type for which we have no mechanistic explanation, while that of VIR-7831 is in the range expected for half-life extended variant mAbs.

The total lung:blood ratio for VIR-7831 antibody on day 3 (0.25) is within the range of values reported elsewhere, e.g., 0.25 and 0.68 [24, 25], which corroborates the value used in clinical dose projections [10]. The PBPK model predicted greater interstitial space:serum ratio (0.55) compared to total predicted lung tissue:serum ratio (0.31) which is related to the antibodies mostly confined to the extracellular space. This predicted interstitial space:serum ratio is also higher than the alveolar epithelial lining fluid:serum ratio (0.02) which reflects the experimentally observed concentration gradient between alveolar epithelial lining fluid (ELF) and serum. The modeled higher interstitial:serum ratio compared to whole lung:serum is consistent with observations from Eigenmann et al. [26].

VIR-7831 has no binding activity except to the spike protein; hence, its tissue distribution and elimination kinetics in uninfected monkeys can be interpreted as being unbiased by its antigen-binding properties, making it an excellent case study in biodistribution of antibodies. As a result, the insight gained can also be useful for mAbs directed against other targets; through physiologically based pharmacokinetic models, we can capture the biodistribution of mAbs in target tissues while adding the expression of membrane bound or soluble targets to capture any TMDD happening in the system. This can be extrapolated to biologics modalities beyond antibodies and could be a useful tool for dose prediction for humans. The same physiologically based pharmacokinetic framework has been shown to describe the tissue distribution and elimination kinetics of a variety of biological modalities in mice, rats, monkeys, and humans [13], covering the essential species repertoire relevant to the global drug discovery efforts. As such, the framework is not necessarily limited to therapeutic modalities but is expected to be applicable to all soluble proteins, endogenous or dosed [17].

The agreement between the three methods used to measure serum concentrations validated the use of PBPK with PET/CT data as the model accommodates both WT mAb and FcRn affinity enhanced mAb. Furthermore, PET measurements are direct, in vivo, minimally invasive, and quantitative, with no need for ex vivo sampling and tissue preparation artifacts. Even though there were generally higher VIR-7831 levels in blood than VIR-7831-WT, similar tissue:blood ratios were observed across tissues between VIR-7831 and VIR-7831-WT with the exception of the liver and the pulmonary bronchi at some time points. As the liver is a primary organ of antibody catabolism, the ^89^Zr-labeled antibody undergoing local degradation would be expected to accumulate in this site, while any free ^89^Zr in serum would be captured in the kidneys [13] or in the bone depending on its complexed composition, as the oxalate and chloride forms could have significant retention in the bone [27].

The lower VIR-7831 liver SUV_mean_ compared to VIR-7831-WT is consistent with FcRn-dependent mAb recycling that is expected to rescue VIR-7831 from lysosomal degradation [8]. This is also supported by radiomics analysis showing VIR-7831 with smaller interquartile range in the liver, indicating the signal was more uniform for VIR-7831 relative to a more punctate signal for VIR-7831-WT. While brain and skeletal muscle displayed relatively low and constant signal, peripheral blood and highly perfused tissues such as the digestive tract, lungs, and bronchi reached high levels after dosing before decaying over time. Within separate sections of respiratory tract, the highest initial signal was detected in the bronchial and nasal regions which are among the most vascularized tissues [28].

The SUV_mean_ in the pulmonary bronchi tended to be higher in both groups compared to that in other tissues in the respiratory tract and uptake was significantly higher in the VIR-7831-treated group compared to that in the VIR-7831-WT-treated group at days 6 and 10. This finding appears to be specific to pulmonary bronchi rather than a general phenomenon of higher VIR-7831 blood concentrations because other highly vascularized tissues in the respiratory tract, such as the whole lung, showed no differences in group mean uptake between mAbs. The tissue:blood ratio reflects the fraction of mAb content in the capillaries, cells, and interstitial spaces of an organ relative to that in blood. In the case of the lungs, this also includes alveolar ELF, which is directly accessible as bronchial alveolar lavage (BAL). In the case of anti-respiratory syncytial virus (RSV) monoclonal antibody (MEDI-524), it was found that both wild-type and half-life extended versions of the mAb displayed statistically indistinguishable BAL:plasma partition, suggesting that higher FcRn-binding affinity of the mAb primarily enhances ELF exposure through elevated plasma concentration and the two compartments are in connection through predominantly passive paracellular exchange [7, 29]. Interestingly, for several organs, the ratio continued to increase even well beyond the mAb distribution phase, suggesting the impact of residualization given that no residualizing signal is expected in the blood. On day 3, the VIR-7831 group mean tissue:blood ratio in the pulmonary bronchi and lung tissue excluding air space was higher than in other respiratory tract tissues. In addition, the 3D levelset analysis showed significantly higher uptake of VIR-7831 compared to VIR-7831-WT in the lung region most proximal to the pulmonary bronchi. This may suggest that higher blood concentrations for VIR-7831 also result in proportionately higher interstitial concentrations in this lung region as the overall tissue:blood ratio is independent of half-life for mAbs [7, 30]. This was confirmed by the PBPK modeling predicting higher concentrations of VIR-7831 in the lung interstitial space and the alveolar epithelial lining fluid which are important with regard to a mAb neutralizing viral entry into cells, and prevent initial infection of epithelial cells, or interstitial cell–cell spread of virus. The higher interstitial concentration was also observed by radiomics with VIR-7831 exhibiting mean-centralized distribution indicating uniform concentration between lung and interstitial spaces [31].

### Limitations

PET/CT study provides an in vivo longitudinal imaging of the whole animal but as one of the aims was to evaluate alveolar concentration of the antibodies, PET does not have the resolution (only ~ 750 μm in this study) required for direct measurement of the 100-nm-thick layer of alveolar epithelial lining fluid (ELF). Other methods as reported elsewhere [26, 30] are based on ex vivo data where tissue samples are excised and blotted to remove surface blood. The samples are typically small, a few hundred milligrams, and some of the tissue blood is simply lost during sample preparation.

In combination with the PET data, the PBPK modeling provides a much more mechanistic and comprehensive model as compared to others. The model is based off 2-pore hypothesis and the parameters for these were fitted using IgG, albumin, and small domain protein. As a result, the model might not be well calibrated for protein in the size range of 50–100 kDa. In addition, the model used here consists of single endosomal compartment for FcRn interactions, thus not taking into account endosomal transit times, or different affinities as pH acidifies from early endosomes to sorting endosomes.

## Conclusion

In conclusion, the biodistribution of VIR-7831 compared to VIR-7831-WT was studied in monkeys and imaged by PET/CT up to day 14 after dosing. ^89^Zr-VIR-7831 had higher uptake in blood and pulmonary bronchi, and lower uptake in the liver, compared to ^89^Zr-VIR-7831-WT. Higher blood concentrations of VIR-7831 over VIR-7831-WT translated to proportionally higher tissue concentrations. The higher tissue concentrations of the half-life extending LS-modified antibody though only statistically significant in the pulmonary bronchi were found to be in agreement with the level of antibody in circulation, which may guide future biologic drug discovery efforts.

## Supplementary Information

Below is the link to the electronic supplementary material.

Supplementary file1 (DOCX 179 KB)
