# Supplementary material for: In vivo biodistribution and pharmacokinetics of sotrovimab, a SARS-CoV-2 monoclonal antibody, in healthy cynomolgus monkeys
Source: Eur J Nucl Med Mol Imaging. 2022 Oct 28;50(3):667–78. doi: 10.1007/s00259-022-06012-3 (PMC9614201; doi:10.1007/s00259-022-06012-3)
Supplement: Supplementary file 1 — Supplementary file1 (DOCX 179 KB) [file 259_2022_6012_MOESM1_ESM.docx]

**SUPPLEMENTAL MATERIAL**

**In vivo biodistribution, and pharmacokinetics of sotrovimab, a SARS-CoV-2 monoclonal antibody**, **in healthy cynomolgus monkey**

**Methods**

**Radiolabeling and Stability of VIR-7831 and VIR-7831-WT antibodies**

LS-bearing antibody VIR-7831 with its unmodified equivalent, VIR-7831-WT were modified with p-SCN-Bn-Deferoxamine, DFO (Macrocyclics) at a 1:1 molar ratio in 0.1 M Na_2_CO_3_ buffer, pH 9 at 37 °C for one hour. Unreacted DFO was removed by passing the DFO-mAb conjugate through a size exclusion column (PD-10 or G-25 columns , SigmaAldrich). Purity of the antibody-DFO conjugates (DFO-mAb) were also confirmed by injecting into analytical High-performance liquid chromatography, HPLC (Agilent 1200 Series, Agilent Technologies) equipped with an analytical Size Exclusion Column, SEC (Superdex 200, 5/150GL , GE Healthcare Life Sciences).  ^89^Zr oxalate solution (in 1.0 M oxalic acid (≥0.037 GBq/nmol, 1 mCi/nmol, University of Wisconsin-Madison, WI, USA) was first neutralized with 2 M Na_2_CO_3_ in 0.5 M HEPES buffer pH 7.0. ^89^Zr was complexed with DFO-mAb at a ratio of 111 MBq/mg of mAb in 0.5 M HEPES buffer pH 7.0 at 37 °C for one hour. The unbound ^89^Zr was removed by size-exclusion chromatography on G-25 columns (SigmaAldrich). The radiochemical purity was determined by analytical SEC equipped with a radio-detector (Dual Scan-RAM, Lablogic Systems Inc) using 100mM sodium phosphate buffer, pH 7.0 containing 0.05% sodium azide at a flow rate of 0.3 mL/min. The stability of ^89^Zr-VIR-7831 and ^89^Zr-VIR-7831-WT were tested by incubation in 10% monkey serum (obtained from a male cynomolgus monkey) and in PBS at 37°C for up to 7 days and analyzed by radio-iTLC strips (Biodex Medical Systems) and radio-detector using a mobile phase of 20 mM citric acid (pH 5.1).

**Human Neonatal Fc-Receptor (FcRn) Binding Assay**

Streptavidin coated clear 96-Well (STAV) Plate with Blocker BSA (ThermoFisher Scientific) was washed three times with PBS/Tween buffer, pH 7.  1 µg/ml biotinylated human FcRn (Acros Biosystem) in PBS was added to the STAV coated plates at 50 µL/well and incubated for two hours at room temperature with shaking (650 rpm). The plate was washed three times with wash buffer, pH 6 (PBS/Tween at pH 6). ^89^Zr-VIR-7831 and ^89^Zr-VIR-7831-WT, VIR-7831 and VIR-7831-WT antibodies were diluted to a concentration range of 1000 to 0.09 ng/mL in PBS pH 6 containing 1% BSA (SigmaAldrich) and added to the FcRn coated plate at 50 µl/well. The plate was incubated at room temperature for one hour with shaking (650 rpm) and washed three times with the wash buffer, pH 6.   Peroxidase AffiniPure F(ab')₂ Fragment Goat Anti-Human IgG, F(ab')₂ fragment specific (Jackson ImmunoResearch) was diluted in assay buffer to a concentration of 0.16 µg/ml and added to the plate at 50 µL/well. The plate was incubated for one hour at room temperature with shaking (650 rpm) and then washed four times with wash buffer, pH 6. The bound peroxidase conjugate was detected with SureBlue™ TMB Microwell Substrate, KPL (Seracare) at 50 µl/well. The reaction was incubated for 14 minutes at room temperature with shaking (650 rpm) in the dark and then quenched by adding 50 µl/well of 1% HCl. The absorbance was read at 450 nm on the Spectramax M2e plate reader. The optical density (OD) values at 450nm vs. antibody concentration were fitted by applying a sigmoidal (log[agonist] vs. response equation (Variable slope, four parameters) in GraphPad Prism 8.1.2 (GraphPad Software, Inc).

**SARS-CoV-2 Spike Protein Receptor Binding Domain (RBD) Binding Assay**

Recombinant Covid-19 spike protein RBS (Humabs) was diluted to 5 µg/ml in PBS (ThermoFisher Scientific), dispensed in a 96-well flat-bottom, High-Bind microplate (Corning) at 25 µl/well and incubated overnight at 4°C or for at least two hours at room temperature with shaking (650 rpm). The plate was then washed twice with the PBS/Tween wash buffer (PBS supplemented with 0.05% Tween-20). An aliquot of 100 µl assay diluent, Blocker Casein in PBS (ThermoFisher Scientific) supplemented with 0.05% Tween-20 was dispensed per well, incubated for one hour at room temperature and then washed twice with the wash buffer. The concentration of VIR-7831-WT, ^89^Zr-VIR-7831-WT, VIR-7831, and ^89^Zr-VIR-7831 antibodies was measured spectrometrically using a NanoDrop OneC (ThermoFisher Scientific). The mAbs were diluted at a concentration range of 5000 to 0.08 ng/ml in the assay diluent and added 25 µl/well to the RBD coated plate. Samples were incubated at room temperature for one hour with shaking (650 rpm). The plate was then washed four times with the wash buffer. Detection of the bound mAb was performed using Goat Anti-Human IgG-AP (Southern Biotech), diluted 1:500 in assay diluent and applied at 25 µl/well for one hour at room temperature with shaking (650 rpm). The plate was then washed four times with the wash buffer. 4-Nitrophenyl phosphate, pNPP( SigmaAldrich) at 1 mg/ml in bicarbonate buffer (Na_2_CO_3_, Sigma-Aldrich 71345; NaHCO_3_, Sigma-Aldrich 71627) was added at a volume of 50 µl/well and the plate was incubated at room temperature for 60 minutes with shaking (650 rpm) in the dark. The absorbance was read at 405 nm (Spectramax M2e, Molecular Devices). The optical density (OD) values at 405 nm vs. antibody concentration was fitted using a sigmoidal (log[agonist] vs. response equation (Variable slope, four parameters) in GraphPad Prism 8.1.2 (GraphPad Software, Inc).

**Experimental design & PET/CT imaging**

On Day 0, monkeys were sedated with 10 mg/kg ketamine by IM injection before IV injection of radiolabeled antibodies. Each monkey received a total dose of 5 mg/kg (labeled and unlabeled mAb), Table S1. In this study, the 5 mg/kg dose replicates what was used in previous pharmacokinetic studies in this species and represents a lower exposure than observed clinically at 500 mg, a dose that was demonstrated to provide protective exposures against susceptible variants in the lung for up to 28 days, [10]. On Days 1, 3, 6, 10, and 14, monkeys were anesthetized with (10 – 15 mg/kg, IM) ketamine for PET/CT image acquisition.  Atropine (0.05 mg/kg SQ) was given as a pre-anesthetic.  An injection of Dexmedetomidine (0.015 – 0.03 mg/kg) IM was given and bland ophthalmic ointment was placed in the eyes to prevent drying.  The animal was transferred to the scanner bed and provided 100% O_2_ (1.5 – 2.0 liters/minute) via face mask. Body temperature was maintained using a heated recirculating water blanket (37 – 41°C) and respiratory rate was monitored using respiratory sensor (SA Instruments). Heart rate, and SPO_2_ were monitored using Masimo Radical-7 Pulse Oximeter (MSO-9500, MASIMO).  Following a full body CT acquisition for attenuation correction and anatomical reference using the following parameters (720 projections; 90kVp tube voltage; 200 ms exposure time; 1:4 binning; helical acquisition), a full body (4 bed positions) PET scan was acquired and reconstructed with the Tera-Tomo 3D algorithm (4 iterations; 8 subsets; 400-600 keV; 0.7 mm^3^ voxel size).

**Drug concentration in tissues, blood and serum from imaging**

Absolute molar concentrations of ^89^Zr-labelled VIR-7831 and VIR-7831-WT mAbs were calculated using the molar activity *a*(Bq/mole) of the dose as given to each animal, calculated according to the following equation:

$$a\left( \frac{Bq}{mole} \right)=\frac{DoseHot(Ci)\cdot3.7E10\left( \frac{\mathrm{Bq}}{\mathrm{Ci}} \right)\cdot1000\left( \frac{\mathrm{mg}}{g} \right)\cdot\mathrm{MW}_{mAb}\left( \frac{g}{\mathrm{mole}} \right)}{\mathrm{DoseCold}\left( \frac{\mathrm{mg}}{\mathrm{kg}} \right)\cdot BW(kg)}$$

$${mAb}_{tissue}\left( M \right)=\frac{{mAb}_{tissue}\left( \frac{Bq}{ml} \right)\cdot1000\left( \frac{\mathrm{mL}}{L} \right)}{a\left( \frac{Bq}{mole} \right)}$$

$${mAb}_{serum}\left( M \right)=\frac{{mAb}_{blood}\left( M \right)}{(1-Hc)}$$

Where *a* is the molar activity (Bq/mole) of the mAb dose as administered to each animal, MWmAb=150000 g/mol is molecular weight of a typical mAb, DoseHot is the ^89^Zr dose (Ci) and DoseCold is the mAb dose (mg/kg). mAb_tissue_$\left( \frac{Bq}{ml} \right)$ is the mAb concentration in the region of interest and Hc is the monkey hematocrit value, set at 0.38 [16] to calculate mAb molar concentration in serum mAb_serum_(M) from the blood concentration value mAb_blood_(M).

Total serum concentrations (both labeled and unlabeled antibody) were measured by Gyrolab immunoassay using capture agents: Biotinylated Goat Anti-Human IgG Monkey Adsorbed (Southern Biotech) and detection reagent: Alexa Fluor 647 Labelled Goat Anti-Human IgG (Southern Biotech). The Gyrolab immunoassay had a highest limit of quantification (HLQ) at 250 µg/mL and the lower limit of quantification (LLQ) at 1 µg/mL. Data was acquired using Gyrolab Control Version 8.1.5.2015, Watson Version 7.6.1.

**Statistical Analysis**

SAS 9.4 TS Level 1M5 was used to model the PET SUV_mean_ and Tissue:Blood ratio data values and to perform statistical tests of mAb group differences at a 0.05 level of significance. Per each of 37 endpoints, a linear mixed-effect model was fit to log-transformed response values, with baseline body weight, day, group, and the day*group interaction as fixed effects; animal pair as a random effect; and an AR(1) error correlation structure per animal with Kenward-Roger denominator degrees of freedom. GraphPad Prism 8.3.0 (GraphPad) was used to generate the graphs. Terminal half-lives for VIR-7831-WT and VIR-7831 were calculated from Day 6 to week 8 serum immunoassay data from a single-exponential decay model by non-linear regression in Matlab 2019a/SimBiology v5.8.4 (Mathworks).

Quantitative tissue distribution analysis of VIR-7831 and VIR-7831-WT was analyzed in terms of absolute concentrations within the framework of cross-species/cross-modality PBPK as described previously [13,17]. The model was adjusted to account for ^89^Zr signal originating from both intact mAb as well as the residualized label of catabolized mAbs. Residualized label was assumed to be fully retained at the site of catabolism. Non-linear regression analysis in Matlab 2019a/SimBiology v5.8.4 (Mathworks) was used to estimate the fractions of dosed VIR-7831 and VIR-7831-WT mAbs that were irreversibly captured in liver and kidneys between the dosing and first PET scanning session.

**Radiomics Analysis for the PET imaging data**

PET radiomics analysis has been used to study the feasibility of using non-invasive image features as a surrogate for cancer phenotypes and to indicate biological abnormalities [14, 18]. In this study, radiomics features were used to distinguish image patterns between the different groups to derive more insights into the PET distribution. Radiomics features were computed using pyradiomics [19] using SUV distribution within each of the individual tissue and organ of interest (region of interest, ROIs). For each ROI, 107 features were computed including 18 intensity features, 14 shape features, and 75 texture features, resulting in 1926 features per animal scan. A subset of radiomic features were analyzed with the aforementioned linear mixed effect model.

**Levelset Analysis of Lung**

Levelset analysis using scikit-fmm [19] was performed to investigate SUV distribution with proximity to major blood supply. As the bronchi anatomically runs closely with pulmonary arterial branches, the bronchi ROI was used as a surrogate for blood supply. For each voxel within the lung, levelset analysis computes the distance to the bronchi to obtain a distance distribution. The distance distribution is evenly separated into three groups (near, intermediate, far), and each voxel is assigned into one of three groups based on their distance to bronchi. The number of groups were determined to best discretize the distance distribution, each group is approximately 1cm in length. The aforementioned linear mixed effect model was also applied in the Levelset analysis.

**Results**

**Fig S1** Stability kinetics of ^89^Zr-VIR-7831 and ^89^Zr-VIR-7831-WT in both buffer and 10% NHP serum over 7 days.

**Fig S2** Group mean tissue to blood ratio measured on days 1, 3, 6, 10, and 14 in female monkeys following a single 5 mg/kg dose of ^89^Zr-VIR-7831.

**Fig S3** Group mean tissue to blood ratio measured on days 1, 3, 6, 10, and 14 in female monkeys following a single 5 mg/kg dose of ^89^Zr-VIR-7831-WT.

| **Animal #** | **Body weight (Kg)** | **mAb** | **Injected dose (mg)** | | **Injected Activity (mCi)** |
| --- | --- | --- | --- | --- | --- |
|  |  |  | **^89^Zr labeled mAb** | **Unlabeled mAb** |  |
| **1** | 4.92 | VIR-7831 | 1 | 23.60 | 2.510 |
| **2** | 3.75 | VIR-7831-WT | 1 | 17.75 | 2.518 |
| **3** | 4.60 | VIR-7831 | 1 | 22.00 | 2.586 |
| **4** | 3.95 | VIR-7831-WT | 1 | 18.75 | 2.540 |
| **5** | 2.75 | VIR-7831 | 1 | 12.75 | 2.894 |
| **6** | 5.00 | VIR-7831-WT | 1 | 24.00 | 2.893 |

**Table S1 Antibody (mAb)** groups and radiolabeled mAb dose. Each monkey received a total dose of 5 mg/kg (labeled and unlabeled mAb).

| Day post injection | Predicted lung tissue (nM) | | Predicted Interstitial space (nM) | | | | Predicted alveolar epithelial lining fluid (nM) | |
| --- | --- | --- | --- | --- | --- | --- | --- | --- |
|  | **VIR-7831** | **VIR-7831-WT** | | **VIR-7831** | **VIR-7831-WT** | **VIR-7831** | | **VIR-7831-WT** |
| Day 1 | **153** | **137** | | **153** | **135** | **3** | | **3** |
| Day 3 | **125** | **109** | | **219** | **188** | **8** | | **7** |
| Day 6 | **116** | **98** | | **221** | **182** | **8** | | **7** |
| Day 10 | **109** | **88** | | **204** | **158** | **8** | | **6** |
| Day 14 | **103** | **80** | | **188** | **137** | **7** | | **5** |

**Table S2** The physiologically-based pharmacokinetics (PBPK) model predicted concentrations for the total lung tissue, the interstitial space, and the alveolar epithelial lining fluid with no model adjustment for VIR-7831 and VIR-7831-WT mAb.

| Day post injection | Predicted lung tissue:serum | | Predicted Interstitial space:serum | | | | Predicted alveolar epithelial lining fluid:serum | |
| --- | --- | --- | --- | --- | --- | --- | --- | --- |
|  | **VIR-7831** | **VIR-7831-WT** | | **VIR-7831** | **VIR-7831-WT** | **VIR-7831** | | **VIR-7831-WT** |
| Day 1 | **0.30** | **0.30** | | **0.30** | **0.30** | **0.01** | | **0.01** |
| Day 3 | **0.31** | **0.32** | | **0.55** | **0.55** | **0.02** | | **0.02** |
| Day 6 | **0.32** | **0.33** | | **0.60** | **0.61** | **0.02** | | **0.02** |
| Day 10 | **0.33** | **0.34** | | **0.61** | **0.62** | **0.02** | | **0.02** |
| Day 14 | **0.33** | **0.36** | | **0.61** | **0.62** | **0.02** | | **0.02** |

**Table S3** The physiologically-based pharmacokinetics (PBPK) model predicted tissue:serum ratios for the total lung tissue, the interstitial space, and the alveolar epithelial lining fluid with no model adjustment for VIR-7831 and VIR-7831-WT mAb.
